# The effect of silkworms (*Bombyx mori*) chitosan on rumen fermentation, methanogenesis, and microbial population in vitro

**DOI:** 10.14202/vetworld.2024.1216-1226

**Published:** 2024-06-08

**Authors:** Yemima Gresia Sagala, Lincah Andadari, Tri Hadi Handayani, Mohammad Miftakhus Sholikin, Ainissya Fitri, Rusli Fidriyanto, Rohmatussolihat Rohmatussolihat, Roni Ridwan, Wulansih Dwi Astuti, Yantyati Widyastuti, Dilla Mareistia Fassah, Indah Wijayanti, Ki Ageng Sarwono

**Affiliations:** 1 Study Program of Nutrition and Feed Science, Graduate School of IPB University, Bogor Indonesia; 2 Research Center for Applied Zoology, National Research and Innovation Agency (BRIN), Cibinong, Indonesia; 3 Research Group of The Technology for Feed Additive and Supplement, Research Center for Animal Husbandry, Research Organization for Agriculture and Food, National Research and Innovation Agency (BRIN), Gunungkidul 55861, Indonesia; 4 Department of Nutrition and Feed Technology, IPB University, Bogor Indonesia

**Keywords:** CH_4_ production, chitosan, fermentation profile, *in vitro*

## Abstract

**Background and Aim:**

Ruminant enteric methane (CH_4_) is one of the largest sources of greenhouse gases that contribute to global warming. To minimize environmental harm caused by ruminants’ CH_4_ production, natural substances can be used to suppress it. Chitosan from crustacean sources had been known to obstruct CH_4_ generation in the rumen. About 18% of silkworm pupae is chitin, but little is known about the impact of silkworm pupae chitosan on rumen methanogenesis. This study investigated the efficacy of the silkworm chitosan extraction method and its impact on rumen fermentation, methanogenesis, and microbial growth *in vitro*.

**Materials and Methods:**

This study employed a randomized complete block design featuring five treatments and four batches for rumen incubation as the blocking factor. In this study, five treatments were implemented: Control (CO) (basal diet with no added chitosan), basal diet with 6% chitosan from the Chinese Silkworm strain 804 (CHI804), basal diet with 6% chitosan from the PS 01 Hybrid Silkworm strain (CHIPS01), basal diet with 6% chitosan from the Hybrid F1 Japanese 102 × Chinese 202 races (CHIJC02), and basal diet with 6% commercial shrimp shell chitosan as the positive control (CHICOMM). The *in vitro* experiments assessed digestibility, pH, total gas generation, CH_4_ production, ammonia nitrogen (NH_3_-N), and short-chain fatty acid levels, along with microbial population. Data were analyzed using a general linear model followed by Duncan’s test when applicable.

**Results:**

A significant effect on dry matter digestibility (DMD), total gas production, CH_4_, NH_3_-N, and rumen microbial populations (Methanogens, *Ruminoccocus albus*, *Ruminoccocus flavefaciens*, *Selonomonas ruminantium*, *Butyrivibrio fibrisolvens*, *Streptoccocus bovis*, *Prevotella* spp., and *Bacteroides* spp.) was observed (p < 0.05). The extracted chitosan (CHIJC02) used in this study exhibited a similar quality to that of commercial chitosan (CHICOMM). CHI804 treatment could reduce gas production, NH_3_-N production, and *B. fibrisolvens* population significantly (p < 0.05), while CHIJC02 could reduce CH_4_ production, methanogen population, acetate (C_2_) production, and increase propionate (C_3_) production significantly (p < 0.05). CHIJC02 and CHICOMM treatments could also increase the population of *R. flavefaciens*, *S. ruminantium*, and *Bacteroides* spp. significantly (p < 0.05). Chitosan addition significantly (p < 0.05) reduced DMD but did not impact organic matter digestibility or pH.

**Conclusion:**

The extracted chitosan mimics commercial chitosan in physico-chemical properties. Chitosan derived from Japanese and Chinese F1 hybrid silkworm strains demonstrated superior capacity for inhibiting CH_4_ generation compared to commercial chitosan. The quality and effects on methanogenesis, rumen fermentation, and rumen microbial populations can differ depending on the origin of chitosan.

## Introduction

The resolution of the global warming issue remains uncertain. Global warming is primarily driven by the emission of greenhouse gases, including chlorofluorocarbon (CFC), carbon dioxide (CO_2_), methane (CH_4_), Ozon (O_3_), nitrous dioxide (N_2_O), and water vapor (H_2_O). In terms of quantity, CH_4_ is the second largest greenhouse gas after carbon dioxide, but it has a potential 28 times greater than CO_2_ because CH_4_ is considered to be more effective in reflecting infrared light [1]. Ruminants emit significant amounts of CH_4_ due to fermentation by rumen microorganisms of feed structural and non-structural carbohydrates, producing H_2_ and CO_2_. These gases are then used by methanogens to form CH_4_ and then will be released into the atmosphere through the process of eructation [2]. Ruminant livestock emits 2.1 Gt of CO_2_ equivalent CH_4_ annually [3]. Methanogenesis affects the environment and decreases feed efficiency [4]. Methane (CH_4_) will result in a loss of 6%–12% gross energy intake (GEI) or 8%–14% digestible energy intake (DEI), which should be used for growth or milk production; the loss of GEI or DEI causes a decrease in feed efficiency of growth [5]. Reducing CH_4_ emissions from enteric fermentation is essential.

Approaches have been devised to cut down CH_4_ emissions. The strategies that can be implemented are farm management, feed strategy, using feed additives as CH_4_ inhibitors, vaccination, and genetic selection [6]. Antibiotic growth promoters (AGP) were once employed as CH_4_ inhibitor agents, but their use is now forbidden due to concerns over antibiotic resistance. In 2014, Indonesia amended Law No 18 of 2009 with Livestock and Health Law No 41, following the European Union’s 2006 ban on AGP. It is essential to use natural supplements to maintain optimal livestock performance and welfare while minimizing CH_4_ emissions [7].

Chitosan is a non-toxic polyglycosamine containing β-(1-4)-2-acetamido-D-glucose and β-(1-4)-2-amino-D-glucose derived from deacetylation of chitin. Addition of 2% chitosan derived from black soldier flies to feed can alter volatile fatty acid (VFA) profiles and increase propionate (C_3_) concentration, decreasing methane (CH_4_) production as indicated by Haryati *et al*. [8, 9]. Belanche *et al*. [10] reported that 5% chitosan could reduce CH_4_ emissions up to 42%. This antimicrobial property directly reduces the population of methanogens. The positively-charged chitosan binds to the negatively-charged bacteria’s surface, leading to increased membrane permeability, peptidoglycan hydrolysis, cell component leakage, and subsequent death [11]. Chitosan can function as a CH_4_ inhibitor agent.

Chitosan for industrial purposes is mainly extracted from crustaceans, especially crabs, shrimps, and shrimp shells waste from the food industry [12]. The chitin content that can be converted into chitosan in crab shells is 15–30% [13], while, in shrimp shells, there is approximately 15–40% chitin [14]. Silkworms, like crustaceans, can provide chitosan. Silkworm pupae are known to contain as much as 18% chitin, which can be deacetylated into chitosan [15]. Humans cultivate silkworms for their silk but discard the pupae. The cocoon processing involves stages such as boiling, reeling, spinning, pressing, and packing. The pupae will no longer be alive after boiling [16]. Pupae weight is about 60–70% of the total weight of whole cocoons [17]. Silkworms provide additional benefits as an alternative chitosan source. The silkworm, with its rich nutritional value and economic cost benefits from being fed food waste, makes insect cultivation an attractive agrarian pursuit [18]. Silkworm cultivation is less land-intensive than agriculture. In addition, silkworm farming contributes to environmental conservation because insect farming exhibits a relatively low carbon footprint [19].

Studies on chitosan from crustaceans as an enteric CH_4_ inhibitor are common, including those by Belanche *et al*. [10], Goiri *et al*. [20], and Goiri *et al*. [21]. However, the role of chitosan from silkworms on enteric CH_4_ inhibition, rumen fermentation, and rumen microbial population remains unexplored. In this study, various silkworm strains were employed as chitosan provider and investigated for their chitosan yield and methanogenesis suppression capacity.

This study aimed to confirm the chosen method’s suitability for producing high-quality chitosan from silkworm pupae and assess its potential for lowering CH_4_ emissions from ruminant *in vitro*.

## Materials and Methods

### Ethical approval

Animal handling protocol was approved by Animal Ethics Committee of the National Research and Innovation Agency (BRIN) (Approval number 08/KE.02/SK/10/2022).

### Study period and location

This research was carried out from March to September 2023 at the Genomic and Environment Laboratory, BRIN, Cibinong, West Java, Indonesia.

### Sample preparation

Three strains of silkworms –Chinese Silkworm strain 804 (CHI804), basal diet with 6% PS 01 Hybrid Silkworm strain (CHIPS01), and Hybrid F1 Japanese 102 × Chinese 202 races (CHIJC02) – were utilized as chitosan sources in this study. The inner layer was removed from the pupae, which were then dried for 48 h at 60°C. Silkworm pupae were gathered from the Ministry of Environment and Forestry of Indonesia’s Center for Standardization of Sustainable Forest Management Instruments. The pupae were stored at –20°C for 24 h after drying. 1 mm sieve was used to screen ground frozen pupae. In the Soxtec 2050 Automatic System (FOSS, Denmark), crude fat from ground pupae was extracted using n-hexane. Chitosan is produced from defatted silkworm pupae meal. The commercially sourced chitosan from shrimp shell (CHICOMM; HiMedia, India) served as the positive control for assessing quality and rumen digestibility.

### Chitosan extraction

The chitosan extraction was carried out using the methods of Kumari *et al*. [22] and Dahmane *et al*. [23]. Silkworm pupae meal was soaked in a 1:10 w/v hydrochloric acid (1M) solution at 27°C for an hour to demineralize it. It was then neutralized by filtering and washing with hot distilled water. The demineralized samples were soaked for 1 h in a 1:25 w/v solution of 1 M sodium hydroxide at 80°C, then filtered and neutralized. The deproteinization process was carried out thrice. The deproteinization product is soaked in 50% hydrogen peroxide (H_2_O_2_) for 2 h at 60°C, filtered, and then neutralized using hot distilled water and acetone. Chitin undergoes deacetylation following filtration. Chitin was soaked in 45% NaOH solution (1:10 w/v) for 6 h at 110°C, filtered, and neutralized for deacetylation. Chitosan is the outcome of the deacetylation process. The percentage yield of chitosan from defatted silkworm pupae meal was calculated.

### Fourier transform infrared (FTIR) and X-ray diffraction (XRD) analysis

The degree of deacetylation (DD) was measured using an FTIR spectrophotometer (Perkin Elmer UATR Two, US) based on the Kumari *et al*. [22] method. Chitosan extract was ground and sieved through mesh 60 and the spectrum was measured at a wavelength of 4000–400 cm^-1^ and a resolution of 4 cm^-1^ with a data interval of 1 cm^-1^. The DD was calculated using the equation:


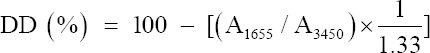


The XRD analysis followed the method of Kumari *et al*. [22]. The sample for XRD analysis was grounded and filtered using sieve mesh 100. Then, the sample was scanned using an X-ray diffractometer (XPert PRO, Netherlands). The diffraction scanner uses Cu radiation with a range of 10°–80° with a size of 0.02, an electric current of 30 mA, a voltage of 40 kV, and a scan speed of 2° min^-1^. The degree of crystallinity was calculated using the equation:

Crystallinity (%) = [(I_110_–I_am_)/I_110_] × 100

### Dry matter (DM) and organic matter (OM) analysis

DM and OM analysis of chitosan were conducted according to Association of Official Analytical Chemists [24].

### Rumen incubation analysis *in vitro*

The method used for rumen incubation *in vitro* was as described by Theodorou *et al*. [25]. Before morning feeding, rumen fluid was collected from four cannulated Brahman cross cattle and filtered using surgical gauze into a pre-warmed flask (39°C). 1 part rumen fluid was mixed with 2 parts McDougall buffer and flushed with CO_2_ gas. 100 mL serum bottle is filled with 0.5 g feed substrate containing 25% Napier grass (*Pennisetum purpureum*) and 75% concentrate. The treatment groups were as follows: basal diet only (CO), basal diet with 6% chitosan from the Chinese Silkworm strain 804 (CHI804), basal diet with 6% chitosan from the PS 01 Hybrid Silkworm strain (CHIPS01), basal diet with 6% chitosan from the Hybrid F1 Japanese 102 × Chinese 202 races (CHIJC02), and basal diet with 6% commercial shrimp shell chitosan (CHICOMM). For 30 s before sealing, CO_2_ was introduced into the serum bottle to preserve anaerobic conditions. The serum bottle was incubated in a 39°C water bath for 48 h. Gas production and its kinetics were determined through 50 mL syringe measurements at specified time intervals (2, 4, 6, 8, 10, 12, 24, and 48 h). CH_4_ emissions were determined based on the proportion of short-chain fatty acids (SCFA) using the methodology of Moss *et al*. [26]:

CH_4_=0.45C_2_–0.275C_3_+0.40C_4_

Where C_2_ is acetate, C_3_ is propionate, and C_4_ is butyrate. The adjusted CH_4_ production emission was calculated based on Jayanegara *et al*. [27] as follows:

CH_4_ after adjustment = CH_4_ before adjustment × 100/H_2_ recovery

The hydrogen recovery value is calculated by the formula from Demeyer and Van Nevel [28]:

Hrec=2Hp/2Hu×100

Where Hrec is hydrogen recovery, Hu is hydrogen utilized, and Hp is hydrogen produced, which was obtained from the following equation:

2Hu=2C_3_+2C_4_+4 CH_4_+C_5,_ and

2Hp=2C_2_+C_3_+4C_4_+2isoC_5_+2C_5_.

Where C_2_ is acetate, C_3_ is propionate, and C_4_ is butyrate iso-C_5_ is iso-valerate and C_5_ is valerate.

After 48 h, the supernatant was separated from the substrate by centrifuging the culture fluid in a 50 mL tube at 5000 rcf for 10 min. The supernatant was frozen at −20°C. The supernatant was analyzed for pH, ammonia nitrogen (NH_3_-N), total SCFA, and microbial population. The substrate was further incubated with 50 mL of pepsin-HCl solution for 48 h and then filtered using Whatman paper no.41. The degradation of DM digestibility (DMD) and OM was measured using this method [29]. Gas production kinetics was determined from López *et al*. [30] equations.

### Ammonia nitrogen analysis

The NH_3_-N analysis was carried out using the method of Souza *et al*. [31]. 10 μL of sample supernatant was added to a test tube for NH_3_-N concentration analysis. 1.5 mL of a phenol solution (50 g L^-1^ phenol, 0.25 g L^-1^ sodium nitroprusside) and 1.5 mL NaOCl solution (16.9 ml L^-1^ NaOCl and 25 g L^-1^ NaOH) were mixed and added to the sample. The reaction tube was incubated in a 39°C water bath for 15 min. The absorbance of the incubated samples was measured using a spectrophotometer set at 630 nm wavelength.

### SCFA analysis

1.25 mL of sample was mixed with 30 mg of 5-sulfosalicylic acid dehydrate and measured for SCFA content using a GC-MS-QP2010 SE (Shimadzu, Japan). The sample was centrifuged at 4°C for 10 min at 23471 rcf. 50:1 solvent: sample ratio and a 3-min cutting time were set for injecting the syringe-collected free particles into the injector. The temperatures of the injection, transfer line, and ion source were all set at 250°C. The column was heated to 100°C, held for 9 min, then heated to 200°C at a rate of 10°C min^-1^ or an additional 10 min. The volatile components were identified and measured using Supelco’s Volatile Free Acid Mix standard (CRM46975, US). This study used the analytical method described by Sarwono *et al*. [32].

### DNA extraction

The QIAamp Fast DNA Stool Mini Kit (Qiagen, Germany) was used to extract rumen microbiome DNA. 48-h rumen culture fluid was used as the sample. The procedure adhered to the manufacturer’s guidelines. The nanophotometer (Implen, Germany) was used to determine the purity and concentration of the DNA extract.). The DNA extract that will be used for microbial dynamic population analysis is a DNA extract that has a concentration of more than 5.5 ng μL^-1^. The DNA extract was stored at −30°C until further analysis.

### Microbial dynamic population analysis

The DNA extract was used to measure the microbial population using real-time polymerase chain reaction (PCR) using comparative cycle threshold (C_T_) method [33], including Methanogens, *Ruminoccocus albus*, *Ruminoccocus flavefaciens*, *Selonomonas ruminantium*, *Butyrivibrio fibrisolvens*, *Streptoccocus bovis* and *Prevotella* spp., and *Bacteroides* spp. The 2^-∆∆CT^ was determined using the C_T_ value difference between the samples and the total bacteria. The 20 μL mixture contains DNA template, SensiFAST SYBR and Fluorescein Kit (Bioline, USA) components, reverse and forward primers, and distilled water. Reverse and forward primers for each target bacteria are mentioned in Table-1. The real-time PCR program consisted of an initial denaturation step at 95°C for 1 min, followed by 40 denaturation cycles at 95°C for 15 s, and a final annealing and extension step at 60°C for 1 min. Methanogens require annealing and extension steps of 30 s each at 60°C.

**Table-1:** Primers used for polymerase chain reaction.

Target	Primer	Sequence (5’ 3’)	Amount (µL)
Total Bacteria	1114-f	CGGCAACGAGCGCAACCC	0.6
	1275-r	CCATTGTAGCACGTGTGTAGCC	0.6
*Ruminococcus albus*	Ra1281f	CCCTAAAAGCAGTCTTAGTTCG	0.5
	Ra1439r	CCTCCTTGCGGTTAGAACA	0.5
*Ruminococcus flavefaciens*	Rflf	GGACGATAATGACGGTACTT	0.9
	Rflr	GCAATCYGAACTGGGACAAT	0.9
Methanogens	q-mcrA-f	TTCGGTGGATCDCARAGRGC	1.2
	q-mcrA-r	GBARGTCGWAWCCGTAGAATCC	1.2
*Selonomonas ruminantium*	SelRum 2F	CAATAAGCATTCCGCCTGGG	0.45
	SelRum 2R	TTCACTCAATGTCAAGCCCTGG	0.45
*Butyrivibrio fibrisolvens*	ButFib 2F	ACCGCATAAGCGCACGGA	0.2
	ButFib 2R	CGGGTCCATCTTGTACCGATAAAT	0.2
*Genus Prevotella*	PrevGen 4F	GGTTCTGAGAGGAAGGTCCCC	0.2
	PrevGen 4R	TCCTGCACGCTACTTGGCTG	0.2
*Genus Bacteroides*	AllBac 296f	GAGAGGAAGGTCCCCCAC	0.2
	AllBac 412r	CGCTACTTGGCTGGTTCAG	3.6
*Streptococcus bovis*	StrBov 2F	TTCCTAGAGATAGGAAGTTTCTTCGG	8.8
	StrBov 2R	ATGATGGCAACTAACAATAGGGGT	8.8

### Statistical analysis

This research used a randomized complete block design with five treatments and four incubation batches as blocks. Data were analyzed using general linear model analysis with the following mathematical model;

Y=β_1_X_1_+β_2_X_2_+β_3_X_3_+ε

Where Y is the observed value, β is the population slope coefficient, X is the independent variable, and ε is the random error term. Significant different effects in results were accepted at the probability level of p < 0.05, then a further test was carried out, namely, the Duncan’s Test using the Statistical Package for the Social Sciences (SPSS) software version 25.0 (IBM Corp., NY, USA).

## Results

### Chitosan qualities

**Figure-1:**
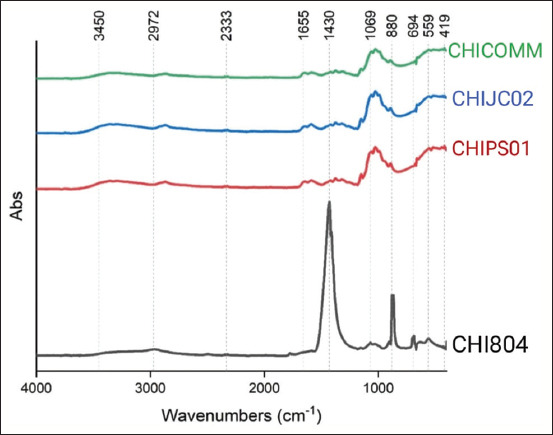
Fourier transform infrared spectra of Chitosan in the wavenumber range 4000–400 cm^-1^. CHI804: Chitosan from the Chinese Silkworm strain 804, CHIPS01: Chitosan from the PS 01 Hybrid Silkworm, CHIJC02: Chitosan Hybrid F1 Japanese and Chinese races 102 × 202, CHICOMM: shrimp shell chitosan (commercial).

**Figure-2:**
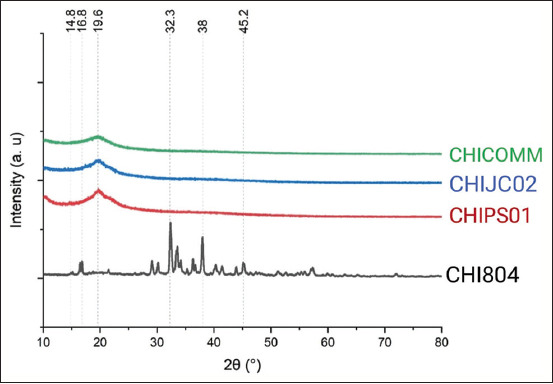
X-ray diffraction spectra of chitosan in 2Ɵ range of 10°–80°. CHI804: Chitosan from the Chinese Silkworm strain 804, CHIPS01: Chitosan from the PS 01 Hybrid Silkworm, CHIJC02: Chitosan Hybrid F1 Japanese and Chinese races 102 × 202, CHICOMM: shrimp shell chitosan (commercial).

Chitosan yield varied from 1.6% to 7.7%, with CHIJC02 producing the least and CHI804 producing the most (Table-2). The absorbance spectra of silkworm pupae chitosan and commercial chitosan are compared in Figure-1 through FTIR with absorbance values between 4000 and 400 cm^-1^. The typical bonds of chitosan were observed at 3354 cm^-1^ (O-H) showed in CHIJC02 and CHICOMM, 2968 cm^-1^ (C-H) showed in CHI804, and 2872 cm^-1^ (CH and CH2 group) showed in CHIPS01, CHIJC02, and CHICOMM. The bonds of chitosan were also observed at 1775 cm^-1^ and 1411 cm^-1^ (C=O) in CHI804, and 1429 cm^-1^ (C=O) showed in CHI804, CHIPS01, and CHIJC02. The other bonds of chitosan were observed at 1376 cm^-1^ (C-H), shown in CHIPS01, CHIJC02, and CHICOMM. C-H stretching was also found at 1069 cm^-1^, 880 cm^-1^, 694 cm^-1^, and 687 cm^-1^, which were shown in CHI804. The typical bonds of chitosan, C-H, were also observed at 1060 cm^-1^ and 893 cm^-1^ which showed in CHIPS01, CHIJC02, and CHICOMM. The C-Cl bonds were observed at 672 cm^-1^–560 cm^-1^ and C-Br bonds observed at 519 cm^-1^ showed in CHIPS01 and CHIJC02. Si-O bonds were also observed at 444 cm^-1^–419 cm^-1^ and 1027 cm^-1^. All of the chitosan samples showed a similar DD result to commercial chitosan. The crystallinity of CHIPS01, CHIJC02, and CHICOMM was indicated by strong peaks at 19.6°, while CHI804 revealed strong peaks at 32.3° and 38° (Figure-2). The percentage of crystallinity in CHI804 chitosan is greater than in other treatments. In addition, water content and ash content analyses were included in the study. Compared to other treatments, CHI804 had the highest water and ash content.

**Table-2:** Chitosan extraction yield and quality.

Parameters	CHI804	CHIPS01	CHIJC02	CHICOMM
Yield (%)	7.75	2.81	1.60	N/A
Deacetylation (%)	98.92	99.13	99.27	99.04
Crystallinity (%)	66.47	28.23	26.31	25.51
Water Content (%)	13.15	8.98	9.28	8.73
Ash (% DM)	70.30	0.20	0.45	1.20

N/A=Data are not available, CHI804=6% chitosan from the Chinese Silkworm strain 804, CHIPS01=Chitosan from the PS 01 Hybrid Silkworm, CHIJC02=Chitosan Hybrid F1 Japanese and Chinese races 102 × 202, CHICOMM=Shrimp shell chitosan (commercial)

### Gas production, gas production kinetics, and CH_4_ production

The addition of chitosan had a significant effect (p < 0.05) on total gas production in treatment CHI804 (Table-3). The decrease of gas production in treatment CHI804 occurred in every hour of gas collection. However, the decrease of gas production also occurred during treatment CHIPS01 at 4 h. CHI804 had a lower total gas production by 7.25% relative to CO. Gas production kinetics calculation showed that maximum gas production (B) and gas production rates (C) in CHI804 decreased significantly (p < 0.05); meanwhile, lags (L) in CHI804 increased significantly (*P* < 0.01). The CH_4_ production in mmol L^-1^ decreased significantly (p < 0.05) in CHIJC02 by 28.19% and increased significantly in CHI804 and CHIPS01 by 26.50% and 19.86%. The adjusted CH_4_ production data indicated that the addition of chitosan could significantly reduce (p < 0.05) adjusted CH_4_ production in treatment CHIJC02 by 28.86% than CO. While in CHI804 and CHIPS01, adjusted CH_4_ production increased by 24.84% and 19.46%, and CHICOMM showed the same value as CO.

**Table-3:** Gas production, gas production kinetics, and CH_4_ production.

Parameters	Treatment					SEM	p-value

	CO	CHI804	CHIPS01	CHIJC02	CHICOMM		
Accumulated gas production in							
2 h (mL)	9.00^b^	6.13^a^	8.13^b^	8.38^b^	8.50^b^	1.67	< 0.001
4 h (mL)	17.38^c^	12.13^a^	16.00^b^	16.50^bc^	16.75^bc^	2.73	< 0.001
6 h (mL)	25.25^b^	18.63^a^	23.75^b^	24.38^b^	24.88^b^	3.41	< 0.001
8 h (mL)	32.50^b^	24.75^a^	31.25^b^	31.63^b^	32.00^b^	3.96	< 0.001
10 h (mL)	41.13^b^	33.38^a^	40.25^b^	40.63^b^	41.00^b^	4.24	< 0.001
12 h (mL)	46.13^b^	39.38^a^	45.50^b^	46.25^b^	46.50^b^	4.25	< 0.001
24 h (mL)	63.38^b^	56.88^a^	62.50^b^	63.25^b^	64.00^b^	3.89	< 0.001
48 h (mL)	77.50^b^	71.88^a^	76.63^b^	77.25^b^	77.75^b^	3.42	< 0.001
Kinetics gas production							
B (mL g^-1^)	79.81^b^	76.27^a^	78.79^b^	79.46^b^	80.00^b^	2.65	0.002
C (mL h^-1^)	0.07^b^	0.06^a^	0.07^b^	0.07^b^	0.07^b^	0.007	< 0.001
L (h)	0.46^a^	0.96^c^	0.67^b^	0.63^ab^	0.62^ab^	0.27	0.001
CH4 production							
CH4 (mmol L^-1^)	6.74^b^	9.17^c^	8.41^c^	4.84^a^	5.18^ab^	1.02	< 0.001
Adjusted CH4 (mmol L^-1^)	5.96^b^	7.93^c^	7.40^c^	4.24^a^	4.55^ab^	0.80	< 0.001

B=Maximum gas production, C=Gas production rates, L=lags, CO=Control, CHI804=6% chitosan from the Chinese Silkworm strain 804, CHIPS01=6% chitosan from the PS 01 Hybrid Silkworm, CHIJC02=6% chitosan Hybrid F1 Japanese and Chinese races 102 × 202, CHICOMM=6% shrimp shell chitosan (commercial) and ^a-c^Means with different superscripts within a row significantly differed (p < 0.05), SEM=Standard error of the mean

### Rumen digestibility and fermentation profile

Chitosan addition could reduce rumen DMD significantly (p < 0.05), where CHIJC02 lowered DMD by 18.26% in comparison to the CO (Table-4). However, chitosan had no significant effect on OM digestibility (OMD) (p < 0.05). The addition of chitosan had no significant effect on pH (p < 0.05) (Table-5). Chitosan could reduce NH_3_-N production significantly (p < 0.05), namely, in treatment CHI804 where NH_3_-N were 35.56% lower than CO. There was a significant increase in total VFA (p < 0.05) in CHI804 and CHIPS01, while CHIJC02 and CHICOMM showed the same total VFA as CO. The addition of chitosan had a significant effect (p < 0.05) on the production of acetate and propionate in the CHIJC02 treatment; acetate production was decreased while propionate production was increased. Acetate and propionate production in CHI804, CHIPS01, and CHICOMM showed the same value as the CO, and there was a significant increase (p < 0.05) of butyrate production in CHIJC02 and CHICOMM by 21.2% and 16.71%. Moreover, there was a significant increase (p < 0.05) of iso-butyrate, iso-valerate, and valerate production in CHI804, CHIPS01, and CHIJC02 sequentially up to 61.81%, 57%, and 36%.

**Table-4:** Rumen digestibility analysis *in vitro*.

Parameters	Treatment					SEM	p-value

	CO	CHI804	CHIPS01	CHIJC02	CHICOMM		
DMD (%)	72.20^a^	59.79^b^	59.10^b^	59.02^b^	59.34^b^	7.88	0.036
OMD (%)	95.06^a^	96.72^a^	95.03^a^	95.01^a^	95.22^a^	1.22	0.154

DMD=Dry matter digestibility, OMD=Organic matter digestibility, CO=Control, CHI804=6% chitosan from the Chinese Silkworm strain 804, CHIPS01=6% chitosan from the PS 01 Hybrid Silkworm, CHIJC02=6% chitosan Hybrid F1 Japanese and Chinese races 102 × 202, CHICOMM=6% shrimp shell chitosan (commercial) and ^a,b^Means with different superscripts within a row significantly differed (p < 0.05), SEM=Standard error of the mean

**Table-5:** Rumen fermentation profiles *in vitro*.

Parameters	Treatment					SEM	p-value

	CO	CHI804	CHIPS01	CHIJC02	CHICOMM		
pH	6.78^a^	6.82^a^	6.80^a^	6.82^a^	6.77^a^	0.07	0.854
NH_3_-N (mg dL^-1^)	18.00^b^	11.60^a^	16.50^b^	15.30^ab^	18.20^b^	4.03	0.018
Total SCFA (mM)	23.33^a^	32.77^b^	29.90^b^	19.27^a^	18.96^a^	8.54	< 0.001
Acetate (%)	68.41^c^	65.63^bc^	66.46^bc^	60.37^a^	63.85^ab^	4.88	0.006
Propionate (%)	19.72^ab^	18.71^a^	20.23^ab^	23.66^c^	21.35^b^	1.46	0.003
Iso-butyrate (%)	0.84^a^	2.19^c^	1.15^ab^	1.40^b^	1.20^b^	0.041	< 0.001
Butyrate (%)	8.77^a^	8.88^a^	9.43^ab^	11.13^c^	10.53^bc^	0.54	0.003
Iso-valerate (%)	1.39^a^	3.24^c^	1.77^ab^	2.26^b^	2.02^b^	0.09	< 0.001
Valerate (%)	0.87^a^	1.36^d^	0.97^ab^	1.19^c^	1.06^bc^	0.01	< 0.001
C_2_/C_3_ ratio	3.48^bc^	3.52^c^	3.28^bc^	2.55^a^	3.03^b^	0.07	0.005

SCFA=Short-chain fatty acid, C_2_/C_3_=Acetate per propionate, CO=Control, CHI804=6% chitosan from the Chinese Silkworm strain 804, CHIPS01=6% chitosan from the PS 01 Hybrid Silkworm, CHIJC02=6% chitosan Hybrid F1 Japanese and Chinese races 102 × 202, CHICOMM=6% shrimp shell chitosan (commercial) and ^a-d^Means with different superscripts within a row significantly differed (p < 0.05), SEM=Standard error of the mean

### Rumen microbiome population

The addition of chitosan could change the population number of rumen microbial *in vitro* (Table-6). The results showed that chitosan had a significant effect (p < 0.05) on the microbial population, namely, *Methanogens*, *R. flavefaciens*, *Bacteroides* spp., *B. fibrisolvens*, and *S. ruminantium*, while the populations of *R*. *albus, Prevotella* spp., and *S*. *bovis* had no significant effect (p > 0.05). *Methanogens* population increased in CHIPS01 and CHICOMM, while, in CHIJC02, it decreased and CHI804 showed no differences to CO. *R*. *flavefaciens, Bacteroides* spp., and *S*. *ruminantium* populations increased in CHIJC02 and CHICOMM, while CHI804 and CHIPS01 showed a similar population as CO. The decrease of *B*. *fibrisolvens* population occurred in CHI804, while in CHIPS01, CHIJC02, and CHICOMM, populations showed no differences to CO.

**Table-6:** Relative density of microbial populations with 2^-∆∆CT^ *in vitro*.

Parameters	Treatment					SEM	p-value

	CO	CHI804	CHIPS01	CHIJC02	CHICOMM		
*Methanogen*	1.01^b^	1.26^bc^	1.39^c^	0.24^a^	1.68^d^	0.54	< 0.001
*Ruminococcus albus*	1.16^a^	1.57^a^	0.83^a^	0.98^a^	1.41^a^	0.62	0.321
*Ruminococcus flavefaciens*	1.01^a^	0.97^a^	1.17^ab^	1.72^bc^	1.87^d^	0.56	0.020
*Bacteroides*	1.01^a^	1.21^a^	1.40^ab^	1.91^c^	1.66^bc^	0.40	0.006
*Butyrivibrion fibrisolvens*	1.02^b^	0.42^a^	1.12^b^	1.35^b^	1.41^b^	0.41	0.004
*Prevotella*	1.21^a^	1.28^a^	1.19^a^	1.41^a^	1.76^a^	0.75	0.848
*Streptococcus bovis*	1.05^a^	1.31^a^	1.17^a^	1.43^a^	1.76^a^	0.65	0.273
*Selenomonas ruminantium*	1.01^a^	1.27^ab^	1.17^ab^	1.62^bc^	1.94^c^	0.43	0.014

CO=Control, CHI804=6% chitosan from the Chinese Silkworm strain 804, CHIPS01=6% chitosan from the PS 01 Hybrid Silkworm, CHIJC02=6% chitosan Hybrid F1 Japanese and Chinese races 102 × 202, CHICOMM=6% shrimp shell chitosan (commercial) and ^a-d^Means with different superscripts within a row significantly differed (p < 0.05), SEM=Standard error of the mean

## Discussion

Humans have cultivated silkworms for producing silk thread since ancient times. This process generates silkworm pupae, rich in chitin, as byproducts. Chitin can produce chitosan when deacetylated partially or completely in solid or dissolved form under alkaline conditions [34]. Various silkworm strains’ pupae are suitable for chitosan production. CHI804 yielded the highest chitosan extract (7.75%) compared to CHIPS01 and CHIJC02 with similar yields. The previous research indicated a 14%–16% yield for chitosan extract derived from silkworm pupae [16, 29]. Although silkworm chitosan yields less than crustacean-derived chitosan (18.33% and 17.20% for shrimp and crab, respectively) [32, 33], it is still a valuable source. The low chitosan content in silkworm pupae is due to their high lipid and protein levels. The low chitosan yield in insects, including this one, can be attributed to their high lipid and protein content [32–34].

The DD of extracted chitosan from silkworms 98.92%–99.27%) approximates those of commercial chitosan (99.04%). Our extracted chitosan from silkworms met the requirement of chitosan for food, cosmetics, and biomedical industries, where the minimum acceptable DD for chitosan is ≥70%, ≥80%, and ≥90%, respectively [35]. The positions of the peaks of CHIPS01, CHIJC02, and CHICOMM in the spectra were the same, but their intensities varied. The absorption peaks’ positions and the intensities of CHI804 differed. The study revealed that CHI804 differed chemically from other chitosans. The chitosan extracted from silkworm pupae of CHIPS01 and CHIJC02 displayed a structural resemblance to commercial chitosan.

According to XRD analysis, CHIPS01 and CHIJC02 chitosan spectra were similar to that of commercial chitosan. Unlike other chitosans in the study, the spectra of CHI804 varied. For chitosan samples CHIPS01, CHIJC02, and CHICOMM, spectra revealed a diffraction peak at 2Ɵ = 19.6°, whereas CHI804 exhibited peaks at 2Ɵ = 32.3° and 38°. The degree of crystallinity, as shown in Table-2, aligned with this discovery. Among the chitosan samples, CHI804 had the greatest degree of crystallinity. CHIPS01 and CHIJC02 chitosans had the same degree of crystallinity as commercial chitosan. The more crystalline a sample is, the less it absorbs. The high degree of crystallinity will decrease the amorphous domains and decrease the absorption ability of the sample [36].

The water content of chitosan, as determined from this study, falls between 8.7% and 13.1% (Table-2). The standard water content of commercial chitosan, according to European Food Safety Authority (EFSA) [37], is ≤10%. CHI804 deviates from standards, while CHIPS01 and CHIJC02 adhere to them and commercial chitosan is in compliance. The ash contents of chitosan CHIPS01 and CHIJC02 meet commercial standards (≤3%) as per EFSA [37], whereas that of chitosan CHI804 is high and may be attributed to an inadequate demineralization process. The successful chitin extraction process can be indicated by the ash content. The study revealed that chitosan from CHIPS01 and CHIJC02 were successfully extracted which was indicated by similar physico-chemical characteristic to commercial chitosan.

Adding CHI804 and CHIPS01 chitosan to the feed inhibited fermentation in the rumen *in vitro*, thereby reducing gas production. The fermentation process of structural carbohydrates in the rumen is indicated by gas production. Chitosan has been demonstrated to suppress gas formation in the rumen [9, 35, 38]. Rumen microbes hydrolyze carbohydrates into simple sugars, producing SCFA as their primary output and emitting CO_2_ and CH_4_ as secondary by-products [35, 36].

The analysis of rumen digestibility validated the reduction in gas production. The addition of chitosan resulted in a decrease in DMD of up to 18.25% across all treatments, with the lowest value observed in CHIJC02. Silkworm chitosan did not affect the OMD significantly. According to Jayanegara *et al*. [39], chitosan inhibited DMD but had no impact on OMD [37, 38, 40, 41]. The antimicrobial activity of chitosan might explain the observed reduction in DMD. The population of *B. fibrisolvens* in CHI804 declined in this study. The rumen bacterium *B. fibrisolvens* plays a significant role in the rumen’s complex carbohydrate breakdown [42]. Reducing *B. fibrisolvens* numbers might impact DMD in the rumen. Chitosan exhibits antimicrobial and antioxidant properties [39, 43]. The antimicrobial properties of chitosan lead to a decrease in fiber fermentation by reducing the population of protozoa and fibrolytic bacteria [44]. The positively charged amino groups contained in chitosan will interact ionically with the negatively charged bacterial surface, thereby causing leakage in the bacterial membrane [45]. Zhang *et al*. [46] reported that chitosan inhibited enzymatic activity in fermentation processes by binding to enzymes.

CHI804 inhibited the maximum gas production (B) and production rate (C), while prolonging the production time lag (L). CHIPS01 and CHIJC02 treatments were similar to CO and CHICOMM in their lack of effectiveness. In the CHI804 treatment, decreased total gas production was caused by the antimicrobial effects of chitosan-inhibiting gas-producing microbes [20]. Delayed gas production from CHI804 might yield less gas than other treatments. The C decreases as the incubation process is prolonged. This is caused by the decreasing number of substrates that can be used as raw materials [47].

Chitosan addition significantly affected CH_4_ production. However, the treatments yielded inconsistent outcomes. Compared to the CO, the CHIJC02 treatment reduced CH_4_ and adjusted CH_4_ production by up to 28.86%. Meanwhile, CHI804 and CHIPS01 increased CH_4_ and adjusted CH_4_ production by 24.84% and 19.46% and CHICOMM showed the same value as CO. The origin of chitosan affects its ability to inhibit methanogenesis. The influence of chitosan’s source, purity, dose, and extraction process on its impact on methanogenesis has been demonstrated in a previous study [9].

Physicochemical properties such as crystallinity can significantly impact its methanogenesis inhibition ability. Among the chitosan samples in this study, CHI804 had the greatest crystallinity. Honma *et al*. [48] discovered that lower chitosan crystallinity enhances chitosan resistance to enzymatic and microbial degradation. The CH_4_ generation was determined based on the stoichiometric link between SCFA and CH_4_. The hydrogen recovery percentage used to calculate adjusted CH_4_ was corrected to the actual value, deviating from the assumed 90% by Moss *et al*. [26]. By adjusting CH_4_ values based on actual hydrogen recovery, Jayanegara *et al*. [27] found that they could reduce the overestimation bias in Moss *et al*.’s [26] CH_4_ calculation.

Chitosan from CHIJC02 exhibited both direct and indirect effects on methanogenesis inhibition, as evidenced in the decrease of methanogen population and the limitation of substrate availability and changes in microbial community composition resulting in reduced CH_4_ production [49]. It was observed that chitosan can reduce the *methanogen* population in the CHIJC02 treatment. A decrease in methanogen numbers results in reduced CH_4_ production in the rumen. Previous findings suggest that the addition of feed additives, including phenolic compounds and oil, reduces methanogen population and inhibits CH_4_ production [42, 44, 50]. CHIJC02’s chitosan reduces CH_4_ production in the rumen by decreasing digestibility and promoting propionate fermentation. In this study, CHIJC02 reduced DMD levels while increasing propionate and decreasing acetate, resulting in a lower C_2_/C_3_ ratio. The C_2_/C_3_ ratio is an indicator of change in the fermentation pattern in the rumen, where the lower C_2_/C_3_ ratio showed a shift toward propionate production [49]. Shifting the fermentation process toward propionate production in the rumen could hinder the H_2_ generation which is essential for CH_4_ formation. The increase in propionate occurred due to an increase in propionate-producing bacteria such as *S*. *ruminantium* and *Bacteroides* spp. [51]. This also affects CH_4_ production due to the lack of H_2_, which is needed to form CH_4_ [52]. According to studies by Zanferari *et al*. [50] and Mingoti *et al*. [53], the addition of chitosan in feed enhances propionate production. Lower propionate production will lower CH_4_ production [54]. In the CHIPS01 and CHICOMM treatments, where methanogen populations grew, there was no corresponding rise in CH_4_ gas output. Decreased digestibility lessens fermentation, hindering methanogens’ CH_4_ generation through reduction of CO_2_ and H_2_ [55].

CHI804 and CHIPS01 silkworm chitosans significantly increased total SCFA. The increase in total SCFA points to heightened carbohydrate fermentation [56]. The high ash content in CHI804, indicative of its elevated mineral content within chitosan, enhances the activity of rumen microbes [57]. The addition of silkworm chitosan had no impact on the total SCFA content of CHIJC02 and CHICOMM. SCFA is the main energy source for ruminants, and the chitosan’s inhibition on digestibility might not affect energy intake from feed. This phenomenon is also found when an indirect methanogenesis inhibition takes place in the rumen by the addition of phloroglucinol [49]. The addition of CHIJC02 and CHICOMM chitosan resulted in decreased acetate production and subsequently increased numbers of *R. flavefaciens* among major cellulolytic bacteria, which primarily produce acetate when co-cultured with methanogen [58]. Despite an increasing population of *R. flavefaciens*, lower acetate production was observed. Latham [58] showed that in the absence of H_2_ utilizing microbe, *R. flavefaciens* produces one or more fermentation products such as ethanol, succinic acid, and/or lactic acid. In the rumen, succinic and lactic acids are metabolized into propionic acid [46, 47]. The increase in propionate production could suggest that *R. flavefaciens* generates succinic acid and/or lactic acid upon addition of CHIJC02 and CHICOMM chitosan.

The rumen’s pH level reflects the continuity of the fermentation process in the rumen *in vitro* media. The addition of chitosan did not significantly alter pH, with the resulting pH ranging from 6.770 to 6.818. The fermentation was in good condition, as indicated by a normal pH value. According to Liu *et al*. [59], the rumen is most stable when its pH falls within the range of 6.2–7.0. Ammonia nitrogen originates from the hydrolysis of protein in the feed. Protein and non-protein nitrogen are hydrolyzed into peptides and amino acids by microbes and will then be degraded into ammonia [60]. In the CHI804 treatment, adding chitosan considerably decreases NH_3_-N production. Less NH_3_-N is generated in the rumen when amino acid breakdown is reduced. The antimicrobial properties of the chitosan contribute to the reduction in NH_3_-N production. The addition of CHI804 chitosan caused a decrease in the population of *B. fibrisolvens*, which significantly contributes to lower protein digestion [61]. Chitosan’s impact on bacterial cell walls’ permeability decreases bacterial populations and reduces fermentation product generation [62]. According to Goiri *et al*. [21], chitosan addition reduced NH_3_-N production, consistent with our findings. Part of the ammonia formed in the rumen will be used for microbial protein synthesis so that the decrease in NH_3_-N production is positively correlated with the growth of the population of *B. fibrisolvens* bacteria as proteolytic bacteria that degrade ammonia [63].

## Conclusion

The extracted silkworm pupae chitosan exhibits the same physico-chemical properties as commercially available chitosan. Chitosan extracted from silkworm pupae suppressed *in vitro* rumen fermentation. The source and physicochemical features of chitosan can affect its inhibitory impact on *in vitro* rumen methanogenesis. Chitosan extracted from Japanese × Chinese Hybrid F1 silkworms directly and indirectly suppressed methanogenesis *in vitro* by decreasing methanogen activity, reducing DMD, and modifying the rumen microbial population. Studies are required to investigate chitosan’s effect on methanogenesis in extended rumen fermentation through *in vivo* or RUSITEC tests. Investigating chitosan’s molecular weight and antioxidant properties is crucial for understanding its rumen methanogenesis inhibition mechanism.

## Authors’ Contributions

YGS, KAS, LA, THH, MMS, AF, RF, RS, RR, WDA, YW, DMF, and IW: Conception and design of the study, conducted the experiments, analyzed the data, and drafted the manuscript. YGS, KAS, THH, and RF: Sample preparation. All authors have read, reviewed, and approved the final manuscript.
